# Clinical prediction in defined populations: a simulation study investigating when and how to aggregate existing models

**DOI:** 10.1186/s12874-016-0277-1

**Published:** 2017-01-06

**Authors:** Glen P. Martin, Mamas A. Mamas, Niels Peek, Iain Buchan, Matthew Sperrin

**Affiliations:** 1 Health e-Research Centre, University of Manchester, Vaughan House, Portsmouth Street, M13 9GB Manchester, UK; 2 Keele Cardiovascular Research Group, Keele University, Stoke-on-Trent, UK; 3 NIHR Greater Manchester Primary Care Patient Safety Translational Research Centre, University of Manchester, Manchester, UK

**Keywords:** Clinical prediction models, Model aggregation, Validation, Computer simulation, Contextual heterogeneity

## Abstract

**Background:**

Clinical prediction models (CPMs) are increasingly deployed to support healthcare decisions but they are derived inconsistently, in part due to limited data. An emerging alternative is to aggregate existing CPMs developed for similar settings and outcomes. This simulation study aimed to investigate the impact of between-population-heterogeneity and sample size on aggregating existing CPMs in a defined population, compared with developing a model de novo.

**Methods:**

Simulations were designed to mimic a scenario in which multiple CPMs for a binary outcome had been derived in distinct, heterogeneous populations, with potentially different predictors available in each. We then generated a new ‘local’ population and compared the performance of CPMs developed for this population by aggregation, using stacked regression, principal component analysis or partial least squares, with redevelopment from scratch using backwards selection and penalised regression.

**Results:**

While redevelopment approaches resulted in models that were miscalibrated for local datasets of less than 500 observations, model aggregation methods were well calibrated across all simulation scenarios. When the size of local data was less than 1000 observations and between-population-heterogeneity was small, aggregating existing CPMs gave better discrimination and had the lowest mean square error in the predicted risks compared with deriving a new model. Conversely, given greater than 1000 observations and significant between-population-heterogeneity, then redevelopment outperformed the aggregation approaches. In all other scenarios, both aggregation and de novo derivation resulted in similar predictive performance.

**Conclusion:**

This study demonstrates a pragmatic approach to contextualising CPMs to defined populations. When aiming to develop models in defined populations, modellers should consider existing CPMs, with aggregation approaches being a suitable modelling strategy particularly with sparse data on the local population.

**Electronic supplementary material:**

The online version of this article (doi:10.1186/s12874-016-0277-1) contains supplementary material, which is available to authorized users.

## Background

Clinical prediction models (CPMs), which compute the risk of an outcome for a given set of patient characteristics, are fundamental to clinical decision support systems. For instance, practical uses of CPMs include facilitating discussions about the risks associated with a proposed treatment strategy, assisting audit analyses and benchmarking post-procedural outcomes. Consequently, there is growing interest in developing CPMs to support local healthcare decisions [1, 2]. Although there might be existing models derived for similar outcomes and populations, it is vital they are appropriately updated, validated and transferred between different contexts of use. Baseline risk and predictor effects may differ across populations, which can cause model performance to decrease when transferring an existing CPM to the local population [3–6]. This between-population-heterogeneity frequently leads to researchers rejecting existing models and developing new ones [5, 7–10]. However, this framework is undesirable because the dataset used to derive the new CPM is often smaller than previous derivation datasets and can lead to multiple models for the same outcome. For instance, over 60 previously published models predict breast cancer [11], which is perplexing and unhelpful to end-users.

As a motivating example, consider a user wishing to predict short-term mortality post cardiac-surgery. There are several existing CPMs available, including the Logistic EuroSCORE (LES), the EuroSCORE II (ESII), the STS score and the German Aortic Valve Model (German AV) [12–16]. These models share some common predictors, for example gender, arterial disease outside the heart and recent heart attack, but some predictors appear only in a subset of the CPMs. For instance, diabetes is only incorporated into the ESII and STS models, while atrial fibrillation is only in the STS and German AV models. Moreover, definitions and coding of some predictors could differ: examples include left ventricular ejection fraction and age.

While differences in variable definitions between existing CPMs add complexity, the prior information encapsulated by each model can be exploited. A generalizable existing CPM could serve as an informative prior for a new population; for example, by transferring information regarding likely covariate-outcome relations, as in stacked regression [9, 17]. However, there has been limited investigation into the impact of sample size and between-population-heterogeneity on the performance of model aggregation versus deriving a new CPM.

This simulation study considers a situation in which there is a new (local) population, with associated data, and interest lies in developing a CPM for it. The modeller must make a choice between utilising existing CPMs that have been developed in different populations, developing a new model and disregarding existing ones, or some mixture of the two [18]. We hypothesised that the modelling strategy that optimised performance would depend on: 1) the degree of variation in risk across multiple populations (between-population-heterogeneity); and 2) the quantity of data available in the local population, relative to the size of previous derivation datasets.

## Methods

Throughout this study, all CPMs will be assumed to be logistic regression models, although the techniques apply to other types of prediction model, such as those for time-to-event outcomes. Stacked regression (SR) [9, 17], principal component analysis (PCA) [19, 20] and partial least squares (PLS) are three possible methods that simultaneously aggregate and calibrate existing models to a new population. We describe SR and PCA here, with PLS described in Additional file 1. This study compares the three aforementioned aggregate approaches with deriving a new model; possible techniques of redevelopment are also outlined in this section.

### Model aggregation: stacked regression

Consider a collection of *M* existing logistic regression CPMs, which all aim to predict the same binary outcome but were derived in different populations, *j* = 1, …, *M*. For a set of observations *i* = 1, …, *n*_*j*_ from population *j*, let ***X***_*j*_ denote the *n*_*j*_ × *P* matrix of predictors that are potentially associated with the outcome, ***Y***_*j*_. Here, *P* represents the number of predictors available across all populations; a predictor that is not present in a given CPM simply has coefficient zero. Then, for *i* = 1, …, *n*_*j*_, the linear predictor (LP) from the *j* ^th^ existing CPM, LP_*i*,*j*_, is given by

$$ {\mathrm{LP}}_{i,j} = {\beta}_{0,j}+{\displaystyle \sum_{p=1}^P}{\beta}_{p,j}{x}_{i,p} $$

with intercept *β*_0,*j*_ and coefficients *β*_1,*j*_, …, *β*_*P*,*j*_, at least one of which is non-zero.

Suppose we then have a new local population, *j* = *M* + 1. Stacked regression aims to weight the *M* linear predictors (calculated for each observation in the new local population) to maximise the logistic regression likelihood. Specifically, SR assumes that for *i* = 1, …, *n*_*M* + 1_, *Y*_*i*,*M* + 1_ ∼ Bernoulli(π_i,M + 1_) where *π*_*i*,*M* + 1_ = *P*(*Y*_*i*,*M* + 1_ = 1) with

$$ \log \left(\frac{\pi_{i,M+1}}{1-{\pi}_{i,M+1}}\right)={\widehat{\gamma}}_0+{\displaystyle \sum_{j=1}^M}{\widehat{\gamma}}_j{\mathrm{LP}}_{i,j} $$

under the constraint that ${\widehat{\gamma}}_1,\dots,\ {\widehat{\gamma}}_M\ge 0$ to account for collinearity between the existing CPMs. Here, LP_*i*,*j*_ denotes the linear predictor from the *j* ^th^ existing CPM calculated for observation *i* ∈ [1, *n*_*M* + 1_] in the new local population. Thus, we have

$$ \log \left(\frac{\pi_{i,M+1}}{1-{\pi}_{i,M+1}}\right) = {\widehat{\gamma}}_0+{\displaystyle \sum_{j=1}^M}{\widehat{\gamma}}_j{\beta}_{0,j} + {\displaystyle \sum_{p=1}^P}{\displaystyle \sum_{j=1}^M}{\widehat{\gamma}}_j{\beta}_{p,j}{x}_{i,p}, $$

which can be used to calculate subsequent risk predictions for a new observation. The hat accent above parameters indicates those that are estimated from the local population data. Specifically, SR estimates ${\widehat{\gamma}}_j$ but not *β*_*p*,*j*_, which are taken as fixed values from the published existing CPM.

### Model aggregation: Principal Components Analysis (PCA) regression

Let **LP** denote the *n*_*M* + 1_ × *M* matrix, with (*i*, *j*)^th^ element being the linear predictor for the *j* ^th^ existing CPM calculated for observations *i* = 1, …, *n*_*M* + 1_ in the local population. The singular value decomposition of **LP** gives an *M* × *M* rotation matrix, **ν**. Multiplying **LP** by **ν,** gives the *n*_*M* + 1_ × *M* matrix of principal components, **Z**. PCA regression again assumes that *Y*_*i*,*M* + 1_ ∼ Bernoulli(*π*_*i*,*M* + 1_) for *i* = 1, …, *n*_*M* + 1_ with

$$ \log \left(\frac{\pi_{i,M+1}}{1-{\pi}_{i,M+1}}\right)={\widehat{\theta}}_0+{\displaystyle \sum_{j=1}^M}{\widehat{\theta}}_j{Z}_{i,j}. $$

Unlike in SR, no restrictions are placed on the parameters ${\widehat{\theta}}_j$ since, by definition, the principal components, **Z**, are uncorrelated. One can obtain predictions for a future observation by converting the above aggregate model onto the scale of the original risk factors,

$$ \log \left(\frac{\pi_{i,M+1}}{1-{\pi}_{i,M+1}}\right)={\widehat{\theta}}_0+{\displaystyle \sum_{j=1}^M}{\widehat{\theta}}_j\left(L{P}_{i,1}{v}_{1,j}+\dots +L{P}_{i,M}{v}_{M,j}\right)={\widehat{\theta}}_0+{\displaystyle \sum_{j=1}^M}{\displaystyle \sum_{r=1}^M}{\widehat{\theta}}_j{v}_{r,j}L{P}_{i,r}. $$

### Model redevelopment

Let ***X***_*M* + 1_ denote the *n*_*M* + 1_ × *P* matrix of predictors in the local population with associated binary outcomes ***Y***_*M* + 1_. Then the redevelopment approaches aim to derive a new CPM of the form

$$ \log \left(\frac{\pi_{i,M+1}}{1-{\pi}_{i,M+1}}\right) = {\widehat{\beta}}_{0,M+1}+{\displaystyle \sum_{p=1}^P}{\widehat{\beta}}_{p,M+1}{x}_{i,p} $$

for *i* = 1, …, *n*_*M* + 1_, model intercept, ${\widehat{\beta}}_{0,M+1}$, and coefficients, ${\widehat{\beta}}_{p,M+1}$, thereby disregarding the existing CPMs. In this study, two strategies of redevelopment were considered; namely, backwards selection using Akaike Information Criterion (AIC) and penalised maximum likelihood estimation (ridge regression). The AIC of a model is defined as 2*k* − 2 log(L), where *k* is the number of estimated parameters and L is the maximum likelihood value. Backwards selection under AIC proceeds by starting with the full model (i.e. all available predictors) and iteratively removing predictors until the model that minimises the AIC is obtained. Conversely, ridge regression estimates the coefficients from the full model by maximising the following penalised log-likelihood function

$$ {l}^{*}\left({\widehat{\beta}}_{M+1}\right)=\left({\displaystyle \sum_{i=1}^{n_{M+1}}}\left\{{y}_i \log \left({\pi}_{i,M+1}\right)+\left(1-{y}_i\right) \log \left(1-{\pi}_{i,M+1}\right)\right\}\right)-\lambda \left({\displaystyle \sum_{p=1}^P}{\left({\widehat{\beta}}_{p,M+1}\right)}^2\right). $$

Thus, the penalty shrinks the model coefficients towards zero, with *λ* controlling the degree of penalisation; cross-validation was used to select *λ* that minimised the deviance function.

### Simulation design: general overview

**Fig. 1:**
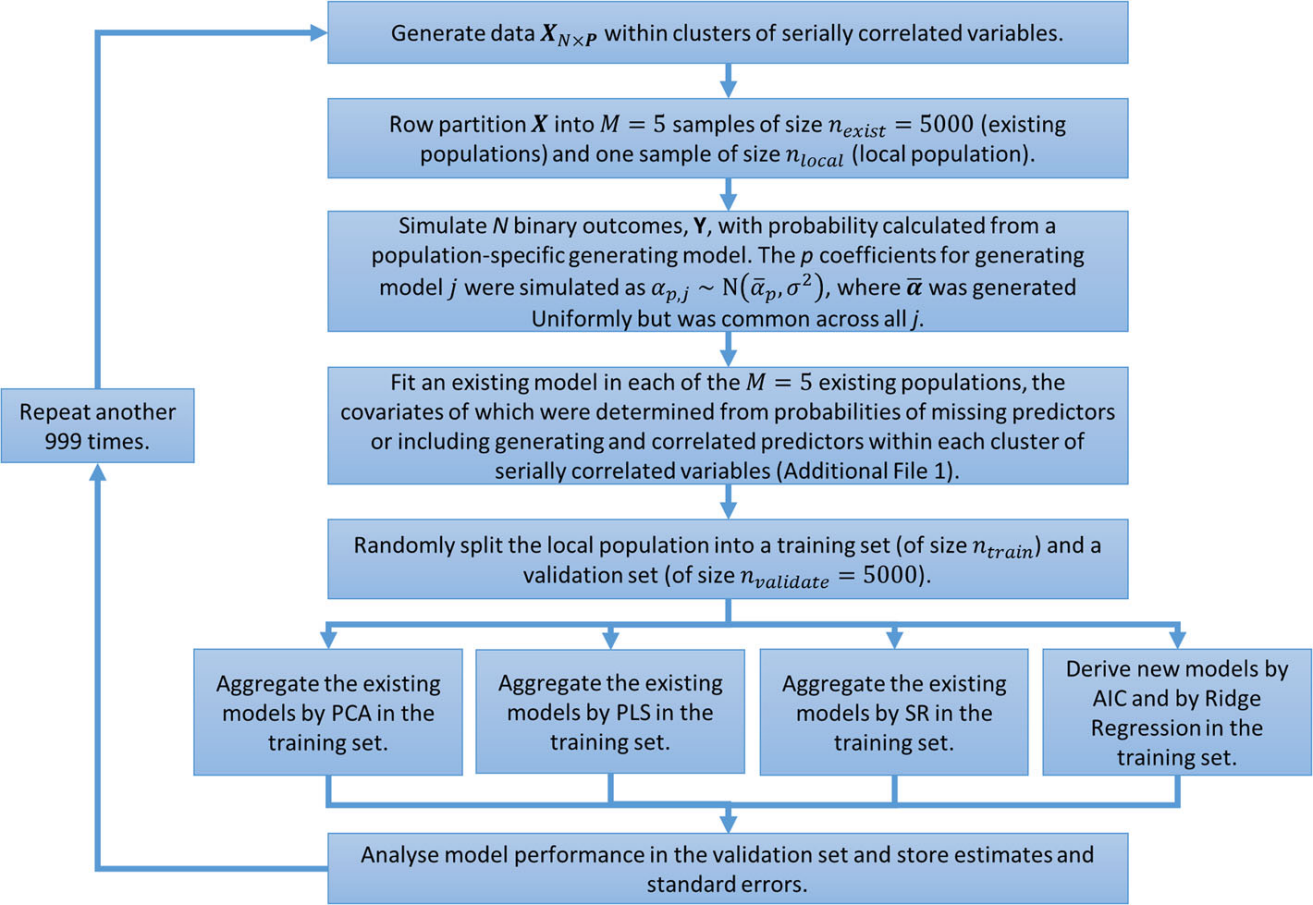
Simulation Procedure: A pictorial representation of the simulation procedure for a given value of population heterogeneity, *σ*, and a given development sample size, *n*_*train*_. This process was then repeated across all combinations of *σ* and *n*_*train*_

Figure 1 visualises the simulation design. The simulation procedure generated both Normally distributed continuous predictors and Bernoulli distributed binary predictors, each within clusters of serially correlated variables to represent multiple risk factors that measure similar patient characteristics. Such data were row partitioned into *M* = 5 distinct subsets of size *n*_*exist*_ = 5000 representing five “existing populations”, and one subset of size *n*_*local*_ representing the “local population”. The *M* = 5 existing populations were each used to fit an existing logistic regression CPMs representing those available from the literature, with each CPM including a potentially overlapping subset of risk predictors (see Additional file 1: Table S1 for details of predictor selection for the existing CPMs). The single local population was randomly split into a training and validation set, of sizes *n*_*train*_ and *n*_*validate*_, respectively (i.e. *n*_*local*_ = *n*_*train*_ + *n*_*validate*_). The training set was used for model aggregation using SR, PCA and PLS in addition to redevelopment using AIC and ridge regression. Datasets frequently only collect a subset of the potential risk factors; to recognise this, exactly those predictors that were included in any of the five existing CPMs were considered candidates during redevelopment. Between simulations *n*_*train*_ was varied through (150, 250, 500, 1000, 5000, 10000); the validation set was reserved only to validate the models with *n*_*validate*_ fixed at 5000 observations. Whilst it is unlikely that local populations would have access to such a large validation set, this was selected here to give sufficient event numbers for an accurate assessment of model performance [21–23]. Additionally, although bootstrapping methods are preferable to assess model performance in real-world datasets, the split-sample method was employed here for simplicity and clear illustration of the methods [24].

Binary responses were simulated in all populations with probability calculated from a population-specific generating logistic regression model, which included a subset of the simulated risk predictors. The coefficients of each population-specific generating model were sampled from a normal distribution, with a common mean across populations and variance σ. Here, higher values of σ induced greater differences in predictor effects across populations and thus represented increasing between-population-heterogeneity. For each of the aforementioned values of *n*_*train*_, simulations were run with *σ* values of (0, 0.125, 0.25, 0.375, 0.5, 0.75, 1).

Across every combination of *σ* and *n*_*train*_, the simulation was repeated over 1000 iterations as a compromise between estimator accuracy and computational time. The simulations were implemented using R version 3.2.5 [25]. The following packages were used in the simulation: “pROC” [26] to calculate the AUC of each model, “plsRglm” [27] to fit the PLS models and the “cv.glmnet” function within the “glmnet” package for deriving a new model by cross-validated ridge regression [28]. The authors wrote all other code, which is available in Additional file 1.

### Simulation design: data-generating mechanisms

In practice, modellers could define any one risk factor through different but potentially related variables and multiple risk factors within a model could be correlated. Hence, the simulation procedure generated risk predictors within clusters of serially correlated variables. Specifically, *P =* 50 predictors were generated within 10 clusters, so that each cluster included *K =* 5 predictors. Predictors had serial correlation within each cluster but were independent between clusters. To represent common real data structures, the simulation generated clusters of binary and continuous predictors in an approximately 50/50 split, with the ‘type’ of each cluster decided at random before each simulation. For simplicity, clusters did not ‘mix’ binary and continuous variables. If ***X***_*N* × *P*_ denotes the *N* × P matrix of predictors (where *N* is the cumulative sample size across all populations) and *ρ* denotes the within-cluster correlation, then the process to generate the predictors was adapted from previous studies [29] as follows:

1. If cluster *κ* includes only continuous predictors then simulate *N* realisations of the predictors at the ‘start’ of the cluster as
  $$ {\boldsymbol{X}}_p\sim \mathrm{Normal}\left(0,1\right), $$
  and simulate the remaining *K-1* correlated predictors as
  $$ {\boldsymbol{X}}_p\sim \rho {\boldsymbol{X}}_{p-1}+\sqrt{\left(1-{\rho}^2\right)}\boldsymbol{\Psi}, $$
  where **Ψ** ∼ Normal(0, 1).
2. Else, if cluster *κ* includes only binary predictors, we generate them as latent Normal. Specifically, simulate *N* realisations of the predictors at the ‘start’ of each cluster as
  $$ {\boldsymbol{X}}_p\sim \mathrm{Normal}\left(0,1\right), $$
  and simulate the remaining *K-1* correlated predictors as
  $$ {\boldsymbol{X}}_p\sim \left\{\begin{array}{c}\hfill {\boldsymbol{X}}_{p-1}\ \mathrm{with}\ \mathrm{prob}.\ \rho \hfill \\ {}\hfill \boldsymbol{\Psi}\ \mathrm{with}\ \mathrm{prob}.\ 1-\rho \hfill \end{array}\right. $$
  where **Ψ** ∼ Normal(0, 1). Each variable in the cluster was then dichotomized to give a pre-defined cluster-specific event rate between 10 and 50%, which are values frequently reported in observational datasets.
3. Repeat steps 1 to 2 across all *κ* = 10 clusters.

Sensitivity analyses across a range of within-cluster correlations, *ρ* ∈ [0, 0.99] showed that the results were qualitatively similar; the results given are for *ρ* = 0.75.

Binary responses for individuals *i* = 1, …, *n*_*j*_ in population *j* were sampled from a population-specific generating logistic regression model with *P*(*Y*_*i*,*j*_ = 1) = *q*_*i*,*j*_, where

$$ \log \left(\frac{q_{i,j}}{1-{q}_{i,j}}\right)={\alpha}_{0,j} + {\displaystyle \sum_{p=1}^{P=50\ }}{\alpha}_{p,j}{\mathrm{x}}_{i,p} $$

with intercept *α*_0,*j*_ and generating coefficients *α*_1,*j*_, …, *α*_50,*j*_. If $\overline{\boldsymbol{\alpha}}$ represents the vector of mean predictor effects across all populations, then the simulation mechanism in each population *j* and generating parameter *p* = 1, …, 50 was

$$ {\alpha}_{p,j}\sim \left\{\begin{array}{cc}\hfill \mathrm{N}\left({\overline{\alpha}}_p,{\upsigma}^2\right)\hfill & \hfill \mathrm{if}\ p\equiv 1\ \left( \mod\ \mathrm{K}=5\right)\hfill \\ {}\hfill 0\hfill & \hfill \mathrm{otherwise}\hfill \end{array}\right. $$

The *p*≡1 (mod K = 5) condition implies (without loss of generality) that in each population, all non-zero generating coefficients were those at the ‘start’ of each cluster. Further, such a simulation procedure induced between-population-heterogeneity by applying random variation to the mean predictor-effects ($\overline{\boldsymbol{\alpha}}$), which was controlled through the value of σ that was introduced above. To represent coefficients frequently reported in published models, $\overline{\boldsymbol{\alpha}}$ was sampled in each simulation as follows:

$$ {\overline{\alpha}}_p\sim \left\{\begin{array}{cc}\hfill \mathrm{Uniform}\left(0.80,\ 1.6\right)\hfill & \hfill \mathrm{if}\ \mathrm{parameter}\ p\ \mathrm{is}\ \mathrm{binary}\hfill \\ {}\hfill \mathrm{Uniform}\left(0.08,\ 0.1\right)\hfill & \hfill \mathrm{if}\ \mathrm{parameter}\ p\ \mathrm{is}\ \mathrm{continuous}\hfill \end{array}\right. $$

In addition, baseline risk undoubtedly differs between populations and, as such, each intercept *α*_0,*j*_ was selected to give an average pre-defined event rate of 20% plus random variation. All simulations were repeated with an event rate of 50%, reflecting a 1-to-1 case-control study. A sensitivity analysis was undertaken where the magnitude of $\overline{\boldsymbol{\alpha}}$ was different across the generating predictors (see Additional file 1 for details), but the results were qualitatively similar as those presented here and so are omitted.

### Simulation design: performance measures

For each iteration within a given simulation scenario, the mean squared error (MSE) between the predicted risk from each aggregate/new model and the actual risk from the generating model were calculated across all samples in the validation set. That is, for model *m* we have, ${\mathrm{MSE}}_{\mathrm{m}}=\frac{1}{n_{validate}}{\displaystyle \sum_{i=1}^{n_{validate}}}{\left({\widehat{\pi}}_{i,m}-{q}_i\right)}^2$, where ${\widehat{\pi}}_{i,m}$ is the predicted risk from model *m* for observation *i* in the validation set and *q*_*i*_ is the generating model risks for this observation. Similarly, the MSE was calculated between the estimated coefficients of each aggregate/new model and the generating coefficients (i.e. ${\mathrm{MSE}}_{\mathrm{m}}=\frac{1}{P}{\displaystyle \sum_{p=1}^P}{\left({\widehat{\beta}}_{p,m}-{\alpha}_{p,M+1}\right)}^2$, where ${\widehat{\beta}}_{p,m}$ is the estimated *p* ^th^ coefficient from model *m* and *α*_*p*,*M* + 1_ is the *p* ^th^ generating coefficient in the local population). Additionally, the calibration and discrimination of each aggregate/new model were calculated in the validation set. The calibration was quantified with a calibration intercept and slope, where values of zero and one respectively represent a well-calibrated model [30]. Discrimination was evaluated by the area under the ROC curve (AUC). All performance measures were averaged across iterations and the empirical standard errors were calculated.

## Results

### Simulated between-population heterogeneity

To gain a practical understanding of the between-population-heterogeneity generated by increasing values of σ, for all simulated parameters the difference between the largest coefficient and smallest coefficient across populations was calculated and summarised (Table 1); such values were compared with corresponding values from the surgical models. Coefficient differences over the LES, ESII, STS and German AV represent heterogeneity across cardiac surgery risk models each developed across multiple countries. Coefficient differences over these models closely matched those generated with σ = 0.25 or σ = 0.375. Conversely, LES and ESII are two models that were developed on very similar cohorts of patients; here, the coefficient differences most closely match those generated by σ = 0.125. Similarly, the average standard deviation of the coefficients across the LES, ESII, STS and German AV models was 0.33 (closely matching σ = 0.375), while that between the LES and ESII was 0.26 (closely matching σ = 0.25). Together, this suggests that σ values between 0 and 0.375 likely represent the majority of clinical situations, with σ values greater than 0.5 arguably rare in practice.

**Table 1:** Summary measures of the difference in generating coefficients values across all simulated populations

	*σ*							LES, ESII, STS, German AV	LES, ESII
	0	0.125	0.25	0.375	0.5	0.75	1		
Lower Quartile^a^	0	0.29	0.58	0.86	1.16	1.74	2.31	0.37	0.14
Median^a^	0	0.31	0.63	0.95	1.27	1.90	2.52	0.57	0.31
Mean^a^	0	0.32	0.63	0.95	1.27	1.90	2.53	0.70	0.37
Upper Quartile^a^	0	0.34	0.68	1.03	1.38	2.06	2.74	0.85	0.54
SD^b^	0	0.12	0.24	0.36	0.48	0.71	0.95	0.33	0.26

The values for the LES, ESII, STS and German AV are approximate, since variable definitions vary between CPMs

^a^: values represent summary measures across all iterations of the average difference between the largest coefficient and smallest coefficient across populations ^b^: the average standard deviation (SD) of each coefficient across all populations. All values aim to guide the between-population heterogeneity induced through different σ values

### Mean square error

For training set samples of 500 or less and when σ ≤ 0.25, all three aggregation approaches resulted in predicted risks that had smaller mean square error and lowered the variance component of the error compared with redevelopment (Additional file 1: Table S2). Similarly, for training sample sizes less than or equal to 500, SR had estimated coefficients with consistently smaller mean square error with lower standard error than the redevelopment approaches, with the exception of the two highest values of σ (Additional file 1: Table S3). Conversely, for training samples of 1000 or more, developing a new model by ridge regression provided parameter estimates with at least equivalent MSE to the aggregation methods.

### Model calibration

**Fig. 2:**
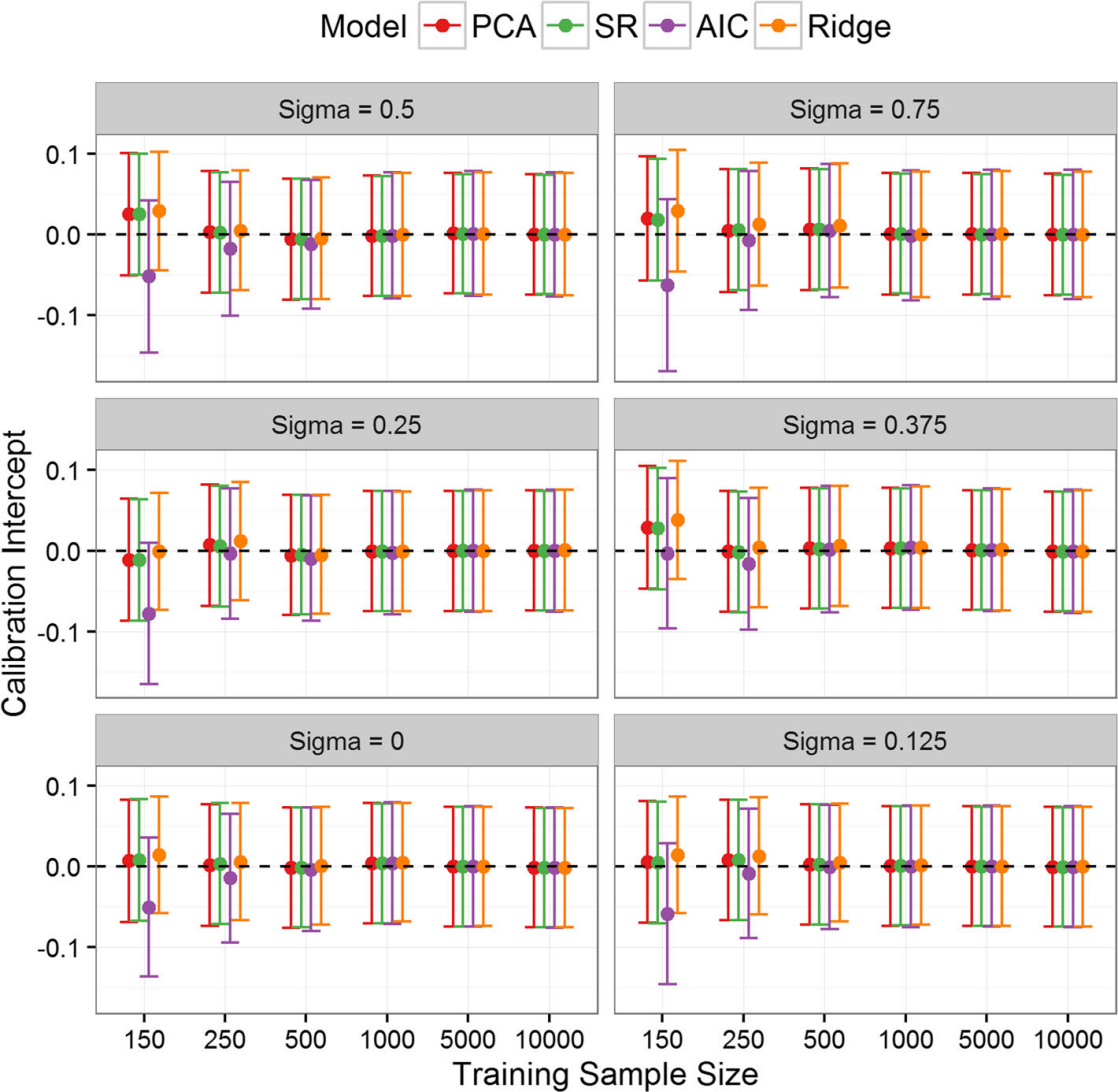
Calibration Intercept: Calibration intercept in the validation set for SR, PCA and the two newly derived models across all simulation situations. The PLS results were nearly identical to SR/PCA and so are omitted for clarity. Note: σ = 1 was removed from the plot for clarity since the results quantitatively similar to σ = 0.75

**Fig. 3:**
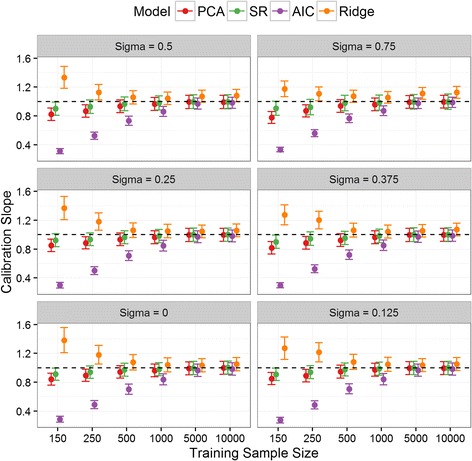
Calibration Slope: Calibration slope in the validation set for SR, PCA and the two newly derived models across all simulation situations. The PLS results were nearly identical to SR/PCA and so are omitted for clarity. Note: σ = 1 was removed from the plot for clarity since the results quantitatively similar to σ = 0.75

The calibration intercepts for all the aggregate/new models were not significantly different from zero in the validation set across all simulations (Fig. 2). Across all values of σ and for training set sizes smaller than 1000, the calibration slope of the AIC derived model was significantly below one indicating overfitting, while that for ridge regression was higher than one, indicating slight over-shrinkage on the parameters (Fig. 3). Conversely, the three aggregate models had a calibration slope not significantly different from one in any scenario, with the exception of PCA in the smallest sample sizes.

**Fig. 4:**
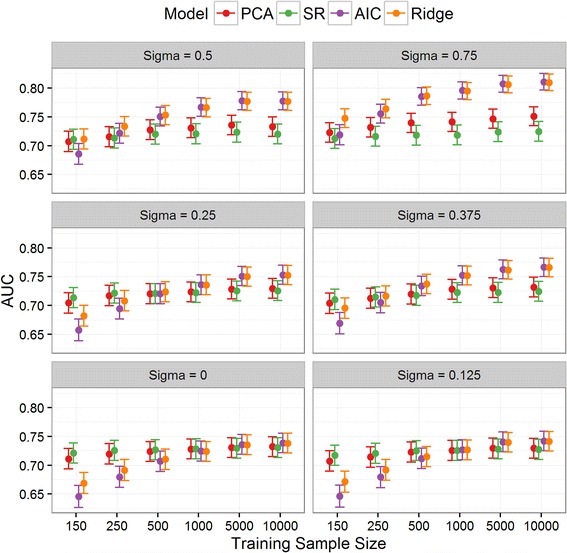
Discrimination: Area under receiver operating characteristic curve (AUC) in the validation set for SR, PCA and the two newly derived models across all scenarios. The PLS results were nearly identical to SR/PCA and so are omitted for clarity. Note: σ = 1 was removed from the plot for clarity since the results quantitatively similar to σ = 0.75

### Model discrimination

When σ ≤ 0.125 and for training sets of 250 or fewer, the AUC of SR was significantly higher than that of both redevelopment approaches (Fig. 4). Although the 95% confidence intervals overlapped, when σ < 0.25 and the training set was less than 1000 observations, the AUC of the two newly derived models (AIC/ridge) were less than that of the aggregate approaches (Additional file 1: Table S4). For instance, when σ = 0, the AUC of SR was higher than that of ridge regression in 988, 968, 821, 498, 56 and 19 out of the 1000 iterations for training set sizes 150, 250, 500, 1000, 5000 and 10000, respectively. Hence, given training set samples of less than 500 and very similar populations, SR provides consistently higher AUC than redevelopment by either ridge or backwards selection.

### Modelling strategy recommendations

A framework that compared modelling strategies of redevelopment and aggregation was developed. For redevelopment, ridge regression was always recommended over AIC since the former more appropriately accounted for low training set sizes. Likewise, all three aggregation approaches performed comparably and so SR was considered here due to the simplicity of implementation. Hence, on comparing ridge regression to SR across all simulation scenarios, if one of the models was well calibrated (calibration intercepts and slopes significantly close to zero and one, respectively) and had significantly higher AUC than the other model, then that modelling strategy was recommended. Conversely, if both models were well calibrated but the AUCs were not significantly different, then a recommendation of “Either” was given. Finally, if one of the models was miscalibrated then the other (calibrated) modelling strategy was recommended.

When the size of the training set was less than 500, then aggregating previously published models by SR was recommended (Table 2). Conversely, developing a new model by ridge regression was recommended in situations where σ > 0.375 and the size of the training set was at least 1000 observations. Between these scenarios, both aggregation methods and redevelopment methods provided indistinguishable performance. Similar recommendations were given when the average event prevalence was increased to 50% (Table 3).

**Table 2:** Modelling strategy recommendations when the mean incidence of adverse outcome was 20%

σ	*n*_*train*_					
	150	250	500	1000	5000	10000
0	SR	SR	Either	Either	Either	Either
0.125	SR	SR	Either	Either	Either	Either
0.25	SR	SR	Either	Either	Either	Either
0.375	SR	SR	Either	Either	Ridge	Ridge
0.5	SR	SR	Either	Ridge	Ridge	Ridge
0.75	SR	SR	Ridge	Ridge	Ridge	Ridge
1.00	SR	SR	Ridge	Ridge	Ridge	Ridge

**Table 3:** Modelling strategy recommendations when the mean incidence of adverse outcome was 50%

σ	*n*_*train*_					
	150	250	500	1000	5000	10000
0	SR	SR	Either	Either	Either	Either
0.125	SR	SR	Either	Either	Either	Either
0.25	SR	SR	Either	Either	Either	Either
0.375	SR	SR	Either	Ridge	Ridge	Ridge
0.5	SR	Ridge	Ridge	Ridge	Ridge	Ridge
0.75	Ridge	Ridge	Ridge	Ridge	Ridge	Ridge
1.00	Ridge	Ridge	Ridge	Ridge	Ridge	Ridge

## Discussion

This study demonstrates that aggregating multiple published CPMs is a useful derivation strategy, particularly when there are limited data available. Stacked regression was a simple yet effective aggregation method, which resulted in predictions and parameter estimates with lowest MSE given low sample sizes and low between-population-heterogeneity. These results are consistent with previous studies [9]. Conversely, AIC derived models were miscalibrated when the training set sample size was between 150 and 500, confirming that small samples lead to overfitting in new regression estimates [8, 31, 32]. Ridge regression, which is a similar concept to parameter shrinkage, mitigated overfitting but was potentially susceptible to slight over-shrinkage. Redevelopment only resulted in a model with better performance than the aggregation methods when there was a large amount of training data or the existing CPMs were significantly heterogeneous.

Previous methodological research around incorporating existing CPMs has focussed on updating a single existing model to the new population of interest [7, 8, 10, 33]. These techniques range from model recalibration to the additional of new risk factors and have been shown to provide improved performance over deriving new prediction models, especially when only small datasets are available [8]. However, updating techniques only adapt one previous model to the current data. In this sense, the concept of model aggregation is analogous to meta-analysis since it aims to synthesise all previous research in the development of the CPM. Moreover, CPMs often perform poorly in high-risk patients. If there are relatively low proportions of high-risk patients in a given population, then the development/ update of a CPM to this population can result in such high-risk, poorly predicted patients becoming more prevalent since parameter estimates occur for the population average. In such situations, one should pay close attention to the residuals of the model; machine-learning methods such as Boosting are a formal approach to this.

Since the aim of this study was to examine the benefits of aggregation over independently deriving a CPM, this study compared each approach separately to solely extract the benefit of either method. However, meta-analysis methods that simultaneously aggregate and redevelop CPMs have been proposed [18, 34, 35]; utilising existing CPMs, expert knowledge and new data optimally requires further research. For instance, risk factors may not be common across existing CPMs, which could lead to bias if one is interested in simultaneously aggregating and redeveloping CPMs in the local population [36]. Previous methodology of CPM meta-analysis with individual patient data has largely been limited to assuming that models share similar risk predictors [10, 18]. Conversely, SR, PCA and PLS relax this assumption [9]. Indeed, the simulation design of this study allowed the existing CPMs to be heterogeneous in their risk predictor set.

Nevertheless, there are potential problems of aggregating CPMs that require attention. Firstly, each existing CPM aims to predict the same outcome and most include very similar subset of predictors, thus inducing a high level of correlation between the multiple CPMs. Although the weights in SR are restricted to be non-negative to avoid situations of negative coefficients caused by inclusion of two correlated models, further work examining the collinearity issues is required [10]. Secondly, differences in risk factor definitions between existing CPMs could potentially weaken the performance of SR, PCA or PLS. The current study aimed to replicate this practical limitation by generating predictors within clusters of correlated variables; here, given a moderate degree of correlation between the multiple similarly defined risk factors, the aggregation methods still performed well. Finally, datasets across populations frequently collect different variables, potentially meaning a variable included in an existing CPM is not available in the local population. In such circumstances of systematically missing covariates, it is unclear how one should calculate the linear predictor for patients in the new local population [37]. If systematically missing risk factors are not handled appropriately, then the aggregate CPM could be biased.

The main strength of this work is that we perform a simulation study under a range of realistic scenarios and consider multiple performance measures, thereby allowing a comprehensive and systematic examination of the aggregation approaches. Conversely, the main limitation is that we simulate only a crude reflection of real between-population-heterogeneity. Over-arching variance of model parameters does not necessarily reflect the complex differences in data-generating processes that may vary between populations. However, without a comprehensive set of joint probability distributions for the covariates of a given model, accurately modelling population heterogeneity is difficult to achieve. Hence, confirmation of our findings will be required from studies in observational datasets. A further limitation is that publication bias is known to impact prognostic research [38], but its effects were not analysed in this study; such bias would lead to overestimation of aggregated regression coefficients. Finally, the aggregation methods assume that each population is a random sample from an over-arching common population. The data-generating mechanisms in this simulation directly matched this assumption by simulating generating model coefficients as a random sample from a common distribution. Similarly, the distributions of the risk predictors were assumed the same between populations.

Overall, the current work suggests a framework of modelling strategy when developing a model for a local/ defined population. In practice, an estimate of the between-population-heterogeneity could be approximated by examining the differences in coefficients between existing CPMs, exploiting clinical knowledge between networks of modelling teams and by examining the distribution of the linear predictors between populations [39]. In many practical scenarios, the variability between populations will be low; thus the situations of *σ* = 0 to *σ* = 0.375 in the current study likely closely represent clinical practice. If the size of the local data is <10% of that the existing CPMs were derived on, and if the multiple populations share clinically similar demographic and procedural characteristics, then we recommend aggregating existing models. Secondly, if the size of local data matches or exceeds that of existing model derivations, then deriving a new CPM could be appropriate, although the existing CPMs could still provide useful prior information about likely covariate-outcome associations. Finally, in all other circumstances, one should consider either aggregation, redevelopment or a combination of the two [18]. Here, the sample sizes relative to the number of predictors per event [31], the estimated population heterogeneity, the quality of the existing CPMs and the availability of variables should drive the chosen method.

## Conclusions

Aggregating existing CPMs is beneficial in the development of a CPM for healthcare predictions in defined populations. In the majority of situations, modellers should consider existing CPMs before developing models anew, with their aggregation potentially providing optimal performance given low sample sizes relative to that of previous model derivations. Deriving a new CPM independent of previous research was only recommended in the unusual situation of having more data available than used to derive existing models, or a local context that is markedly different to those of existing CPMs.

## Additional file

Additional file 1: Details of the Partial Least Squared regression methodology, additional details of the simulation design, additional tables and the simulation R code. (DOCX 49 kb)

## Abbreviations

CPM: Clinical prediction model
ESII: EuroSCORE II
German AV: German Aortic Valve Model
LES: Logistic EuroSCORE
MSE: Mean Square Error
PCA: Principal component analysis
PLS: Partial least squares
SR: Stacked regression
