# Supplementary material for: Clinical prediction in defined populations: a simulation study investigating when and how to aggregate existing models
Source: BMC Med Res Methodol. 2017 Jan 6;17:1. doi: 10.1186/s12874-016-0277-1 (PMC5217317; doi:10.1186/s12874-016-0277-1)
Supplement: Additional file 1: — Details of the Partial Least Squared regression methodology, additional details of the simulation design, additional tables and the simulation R code. (DOCX 49 kb) [file 12874_2016_277_MOESM1_ESM.docx]

Supplementary Material

**Clinical Prediction in Defined Populations: a simulation study investigating when and how to aggregate existing models**

Glen P. Martin, Mamas A. Mamas, Niels Peek, Iain Buchan, Matthew Sperrin

# Supplementary Methods

## Partial Least Squares (PLS) Regression

The notation in this section is in keeping with that from the main paper. PLS regression is similar to Principal Component Analysis (PCA) regression. However, unlike PCA regression, in PLS regression the principal components are derived using both the outcome, **Y**, and the covariates, **LP**. Specifically, PLS simultaneously decomposes **LP** and **Y** in such a way as to explain as much of the covariance between the outcome and the predictors. This contrasts with PCA, where the components are chosen to explain **LP** only. Further computational details can be found in the literature [1, 2] with most statistical software implementing PLS regression.

The resulting components from the PLS are used in the aggregate model in a similar way to that described for PCA regression. Specifically, the form of the aggregate model and the way in which to calculate future predictions follow similar form and reasoning to the details given for PCA. The potential advantage of PLS over PCA is that outcome-covariate associations may be held more strongly in PLS, since the principal components are calculated by considering both the linear predictors and the outcome.

## Simulation Design: existing CPMs predictor selection

Between existing CPMs, some predictors might be missed during modelling or data collection. To replicate this, the predictors used for each existing CPM were randomly selected per iteration based on pre-defined cluster- and model-specific probabilities of: (i) missing all predictors within a cluster, (ii) including a predictor that was correlated with the generating-predictor in a cluster, or (iii) including both the generating-predictor and a predictor correlated with the generating-predictor (**eTable 1**). A scenario of only including a generating-predictor within a cluster was not considered so that an existing CPM predictor was never defined to exactly match the generating model. Additionally, two of the existing CPMs included higher probabilities of missing predictors, thereby representing variation in the existing CPMs performance and development.

**Simulation Sensitivity Analyses**

A range of sensitivity analyses were conducted, including varying the within-cluster correlation ($\rho$) across values of 0.25, 0.5, 0.75 and allowing the magnitude of the mean predictor-effects ($\bar{\boldsymbol{\alpha}}$) to be different across the generating predictors. The set-up for this latter sensitivity analysis was to randomly choose (before the simulation) different Uniform parameters for a range of values across the generating predictors. Specifically, sample $\bar{\boldsymbol{\alpha}}$ as follows:

$$\bar{\alpha}_{p}\sim\left\{ \begin{matrix} Uniform([0.40, 0.80], [0.80, 1.60]) & if parameter p is binary \\ Uniform([0.02,0.08], [0.08, 0.10]) & if parameter p is continuous \end{matrix} \right.$$

where [.,.] denotes the range of possible values. Such Uniform parameters were chosen to mimic coefficients frequently reported in published models.

# Supplementary Tables

**eTable 1:** The probabilities in each candidate model and cluster of serially correlated predictors, used in determine which covariates each existing CPM included.

|  | Serially Correlated Cluster | | | | | | | | | |
| --- | --- | --- | --- | --- | --- | --- | --- | --- | --- | --- |
|  | 1 | 2 | 3 | 4 | 5 | 6 | 7 | 8 | 9 | 10 |
| Existing CPM 1-3 |  |  |  |  |  |  |  |  |  |  |
| P(Miss) | 0.00 | 0.00 | 0.00 | 0.00 | 0.00 | 0.35 | 0.35 | 0.50 | 1.00 | 1.00 |
| P(Correlated Only) | 0.00 | 0.00 | 0.25 | 0.25 | 0.50 | 0.50 | 0.50 | 0.50 | 0.00 | 0.00 |
| P(Correlated AND Generating) | 1.00 | 1.00 | 0.75 | 0.75 | 0.50 | 0.15 | 0.15 | 0.00 | 0.00 | 0.00 |
| Existing CPM 4-5 |  |  |  |  |  |  |  |  |  |  |
| P(Miss) | 0.00 | 0.00 | 0.00 | 0.15 | 0.25 | 0.35 | 0.35 | 0.85 | 1.00 | 1.00 |
| P(Correlated Only) | 0.00 | 0.00 | 0.50 | 0.60 | 0.50 | 0.50 | 0.50 | 0.15 | 0.00 | 0.00 |
| P(Correlated AND Generating) | 1.00 | 1.00 | 0.50 | 0.25 | 0.25 | 0.15 | 0.15 | 0.00 | 0.00 | 0.00 |

**eTable 2:** Mean Square Error (empirical standard error) in the predicted risks from each model and the generating risks from the generating model in the validation population. Bold items indicate the minimum mean square error across each sample size.

| **Model** | **Training set Sample Size** | **σ=0.00** | **σ=0.125** | **σ=0.250** | **σ=0.375** | **σ=0.500** | **σ=0.750** | **σ=1.00** |
| --- | --- | --- | --- | --- | --- | --- | --- | --- |
| PCA | 150 | 0.028 (0.006) | 0.029 (0.007) | 0.032 (0.007) | 0.035 (0.009) | 0.038 (0.010) | 0.045 (0.012) | 0.052 (0.015) |
| PLS | 150 | 0.027 (0.006) | 0.028 (0.006) | 0.031 (0.007) | 0.034 (0.009) | **0.037 (0.010)** | 0.046 (0.012) | 0.053 (0.015) |
| SR | 150 | **0.026 (0.006)** | **0.027 (0.006)** | **0.030 (0.007)** | **0.033 (0.008)** | **0.037 (0.010)** | 0.046 (0.012) | 0.054 (0.015) |
| AIC | 150 | 0.058 (0.018) | 0.060 (0.018) | 0.060 (0.018) | 0.062 (0.019) | 0.063 (0.021) | 0.067 (0.024) | 0.071 (0.026) |
| Ridge | 150 | 0.033 (0.006) | 0.033 (0.006) | 0.034 (0.007) | 0.036 (0.008) | **0.037 (0.009)** | **0.040 (0.011)** | **0.044 (0.013)** |
| PCA | 250 | 0.025 (0.005) | 0.026 (0.005) | **0.028 (0.007)** | 0.032 (0.008) | 0.035 (0.010) | 0.042 (0.012) | 0.048 (0.014) |
| PLS | 250 | 0.025 (0.005) | 0.026 (0.005) | **0.028 (0.007)** | 0.032 (0.008) | 0.036 (0.010) | 0.042 (0.012) | 0.049 (0.014) |
| SR | 250 | **0.024 (0.005)** | **0.025 (0.005)** | **0.028 (0.006)** | **0.031 (0.008)** | 0.035 (0.010) | 0.044 (0.013) | 0.052 (0.014) |
| AIC | 250 | 0.039 (0.009) | 0.039 (0.009) | 0.040 (0.009) | 0.040 (0.010) | 0.041 (0.011) | 0.043 (0.013) | 0.045 (0.015) |
| Ridge | 250 | 0.029 (0.005) | 0.030 (0.006) | 0.030 (0.006) | **0.031 (0.007)** | **0.032 (0.009)** | **0.035 (0.011)** | **0.038 (0.013)** |
| PCA | 500 | **0.023 (0.005)** | **0.024 (0.005)** | 0.027 (0.006) | 0.030 (0.008) | 0.033 (0.009) | 0.040 (0.012) | 0.046 (0.015) |
| PLS | 500 | **0.023 (0.005)** | **0.024 (0.005)** | 0.027 (0.006) | 0.030 (0.008) | 0.033 (0.009) | 0.040 (0.012) | 0.047 (0.015) |
| SR | 500 | **0.023 (0.005)** | **0.024 (0.005)** | 0.027 (0.006) | 0.030 (0.008) | 0.034 (0.009) | 0.043 (0.013) | 0.051 (0.016) |
| AIC | 500 | 0.028 (0.006) | 0.028 (0.006) | 0.029 (0.006) | 0.029 (0.008) | 0.030 (0.009) | 0.032 (0.011) | 0.034 (0.014) |
| Ridge | 500 | 0.026 (0.005) | 0.025 (0.005) | **0.026 (0.006)** | **0.027 (0.007)** | **0.028 (0.008)** | **0.030 (0.011)** | **0.033 (0.013)** |
| PCA | 1000 | **0.022 (0.004)** | 0.023 (0.005) | 0.026 (0.006) | 0.029 (0.007) | 0.032 (0.009) | 0.039 (0.012) | 0.045 (0.015) |
| PLS | 1000 | **0.022 (0.004)** | 0.023 (0.005) | 0.026 (0.006) | 0.029 (0.008) | 0.032 (0.009) | 0.039 (0.012) | 0.045 (0.015) |
| SR | 1000 | **0.022 (0.004)** | 0.023 (0.005) | 0.026 (0.006) | 0.030 (0.008) | 0.034 (0.009) | 0.043 (0.012) | 0.050 (0.015) |
| AIC | 1000 | 0.023 (0.005) | 0.023 (0.005) | **0.024 (0.006)** | 0.025 (0.007) | **0.025 (0.008)** | **0.027 (0.011)** | **0.030 (0.013)** |
| Ridge | 1000 | 0.023 (0.004) | 0.023 (0.005) | **0.024 (0.006)** | **0.024 (0.007)** | **0.025 (0.008)** | **0.027 (0.011)** | **0.030 (0.013)** |
| PCA | 5000 | 0.021 (0.005) | 0.022 (0.005) | 0.025 (0.006) | 0.027 (0.007) | 0.030 (0.009) | 0.037 (0.012) | 0.044 (0.014) |
| PLS | 5000 | 0.021 (0.005) | 0.022 (0.005) | 0.025 (0.006) | 0.027 (0.007) | 0.030 (0.009) | 0.037 (0.012) | 0.044 (0.014) |
| SR | 5000 | 0.022 (0.005) | 0.023 (0.005) | 0.025 (0.006) | 0.029 (0.008) | 0.032 (0.009) | 0.041 (0.013) | 0.050 (0.015) |
| AIC | 5000 | **0.020 (0.005)** | **0.020 (0.005)** | **0.020 (0.006)** | **0.021 (0.007)** | **0.022 (0.008)** | **0.024 (0.011)** | **0.026 (0.013)** |
| Ridge | 5000 | **0.020 (0.005)** | **0.020 (0.005)** | **0.020 (0.006)** | **0.021 (0.007)** | **0.022 (0.008)** | **0.024 (0.011)** | 0.027 (0.013) |
| PCA | 10000 | 0.021 (0.004) | 0.022 (0.005) | 0.025 (0.006) | 0.028 (0.008) | 0.031 (0.009) | 0.036 (0.012) | 0.043 (0.014) |
| PLS | 10000 | 0.021 (0.004) | 0.022 (0.005) | 0.025 (0.006) | 0.028 (0.008) | 0.031 (0.009) | 0.036 (0.012) | 0.043 (0.014) |
| SR | 10000 | 0.022 (0.004) | 0.023 (0.005) | 0.025 (0.006) | 0.029 (0.008) | 0.033 (0.010) | 0.041 (0.013) | 0.049 (0.015) |
| AIC | 10000 | **0.020 (0.004)** | **0.020 (0.005)** | **0.020 (0.006)** | **0.021 (0.007)** | **0.022 (0.008)** | **0.022 (0.011)** | **0.025 (0.013)** |
| Ridge | 10000 | **0.020 (0.004)** | **0.020 (0.005)** | **0.020 (0.006)** | **0.021 (0.007)** | **0.022 (0.008)** | 0.023 (0.011) | 0.026 (0.012) |

**eTable 3:** Mean square error (empirical standard error) in the estimated coefficients from each model and the generating coefficients from the generating model in the validation population. Bold items indicate the minimum mean square error across each sample size.

| **Model** | **Training set Sample Size** | **σ=0.00** | **σ=0.125** | **σ=0.250** | **σ=0.375** | **σ=0.500** | **σ=0.750** | **σ=1.00** |
| --- | --- | --- | --- | --- | --- | --- | --- | --- |
| PCA | 150 | 0.214 (0.067) | 0.215 (0.065) | 0.235 (0.076) | 0.259 (0.098) | 0.288 (0.117) | 0.388 (0.187) | 0.501 (0.266) |
| PLS | 150 | 0.204 (0.059) | 0.207 (0.061) | 0.228 (0.074) | 0.252 (0.096) | 0.281 (0.118) | 0.392 (0.190) | 0.510 (0.272) |
| SR | 150 | **0.192 (0.056)** | **0.196 (0.058)** | **0.218 (0.072)** | **0.241 (0.091)** | **0.271 (0.114)** | **0.374 (0.183)** | **0.492 (0.268)** |
| AIC | 150 | 2.564 (3.387) | 3.025 (3.997) | 2.775 (3.584) | 3.113 (3.947) | 3.237 (4.004) | 3.278 (3.641) | 3.825 (3.860) |
| Ridge | 150 | 0.298 (0.077) | 0.297 (0.077) | 0.313 (0.088) | 0.321 (0.108) | 0.341 (0.126) | 0.414 (0.191) | 0.497 (0.254) |
| PCA | 250 | 0.201 (0.058) | 0.205 (0.059) | 0.220 (0.071) | 0.252 (0.094) | 0.279 (0.120) | 0.370 (0.182) | 0.481 (0.260) |
| PLS | 250 | 0.199 (0.057) | 0.203 (0.057) | 0.218 (0.071) | 0.250 (0.094) | 0.278 (0.120) | 0.374 (0.184) | 0.490 (0.265) |
| SR | 250 | **0.188 (0.055)** | **0.194 (0.056)** | **0.210 (0.069)** | **0.242 (0.091)** | **0.270 (0.119)** | **0.366 (0.184)** | 0.482 (0.263) |
| AIC | 250 | 1.372 (1.547) | 1.364 (1.519) | 1.489 (1.835) | 1.391 (1.336) | 1.579 (1.693) | 1.780 (1.730) | 1.948 (1.395) |
| Ridge | 250 | 0.268 (0.068) | 0.268 (0.070) | 0.274 (0.079) | 0.294 (0.099) | 0.304 (0.120) | 0.371 (0.176) | **0.448 (0.240)** |
| PCA | 500 | 0.194 (0.052) | 0.195 (0.053) | 0.212 (0.067) | 0.240 (0.088) | 0.279 (0.114) | 0.375 (0.184) | 0.491 (0.264) |
| PLS | 500 | 0.193 (0.052) | 0.196 (0.054) | 0.212 (0.067) | 0.240 (0.087) | 0.281 (0.115) | 0.376 (0.185) | 0.496 (0.266) |
| SR | 500 | **0.187 (0.051)** | **0.191 (0.053)** | **0.207 (0.066)** | **0.235 (0.086)** | **0.276 (0.114)** | 0.375 (0.185) | 0.497 (0.268) |
| AIC | 500 | 1.013 (0.162) | 1.031 (0.186) | 1.049 (0.227) | 1.093 (0.255) | 1.175 (0.323) | 1.347 (0.455) | 1.569 (0.641) |
| Ridge | 500 | 0.235 (0.057) | 0.234 (0.059) | 0.239 (0.069) | 0.251 (0.087) | 0.277 (0.107) | **0.333 (0.163)** | **0.412 (0.229)** |
| PCA | 1000 | 0.183 (0.047) | 0.192 (0.051) | 0.209 (0.067) | 0.240 (0.087) | 0.276 (0.117) | 0.372 (0.181) | 0.488 (0.269) |
| PLS | 1000 | 0.183 (0.047) | 0.192 (0.051) | 0.209 (0.067) | 0.241 (0.087) | 0.277 (0.117) | 0.374 (0.182) | 0.491 (0.270) |
| SR | 1000 | **0.181 (0.046)** | **0.189 (0.051)** | **0.207 (0.066)** | 0.239 (0.087) | 0.275 (0.117) | 0.374 (0.181) | 0.498 (0.272) |
| AIC | 1000 | 0.963 (0.142) | 0.967 (0.149) | 0.990 (0.180) | 1.053 (0.232) | 1.104 (0.291) | 1.256 (0.412) | 1.456 (0.557) |
| Ridge | 1000 | 0.205 (0.050) | 0.210 (0.055) | 0.217 (0.066) | **0.232 (0.083)** | **0.249 (0.107)** | **0.306 (0.157)** | **0.388 (0.229)** |
| PCA | 5000 | 0.183 (0.047) | 0.186 (0.049) | 0.205 (0.067) | 0.228 (0.083) | 0.264 (0.113) | 0.365 (0.179) | 0.482 (0.265) |
| PLS | 5000 | 0.183 (0.047) | 0.186 (0.049) | 0.205 (0.067) | 0.229 (0.083) | 0.264 (0.114) | 0.366 (0.179) | 0.483 (0.266) |
| SR | 5000 | **0.183 (0.047)** | 0.186 (0.049) | 0.205 (0.067) | 0.229 (0.083) | 0.265 (0.114) | 0.371 (0.182) | 0.496 (0.271) |
| AIC | 5000 | 0.930 (0.121) | 0.934 (0.136) | 0.961 (0.170) | 0.989 (0.218) | 1.054 (0.272) | 1.231 (0.387) | 1.430 (0.563) |
| Ridge | 5000 | **0.183 (0.048)** | **0.183 (0.050)** | **0.189 (0.066)** | **0.201 (0.077)** | **0.221 (0.103)** | **0.282 (0.152)** | **0.366 (0.227)** |
| PCA | 10000 | 0.183 (0.046) | 0.188 (0.050) | 0.206 (0.065) | 0.236 (0.088) | 0.270 (0.112) | 0.350 (0.175) | 0.472 (0.259) |
| PLS | 10000 | 0.183 (0.046) | 0.188 (0.050) | 0.206 (0.065) | 0.236 (0.088) | 0.270 (0.112) | 0.350 (0.175) | 0.472 (0.259) |
| SR | 10000 | 0.183 (0.045) | 0.188 (0.050) | 0.206 (0.065) | 0.237 (0.088) | 0.273 (0.113) | 0.357 (0.177) | 0.486 (0.265) |
| AIC | 10000 | 0.930 (0.124) | 0.930 (0.135) | 0.963 (0.172) | 1.012 (0.214) | 1.053 (0.274) | 1.201 (0.385) | 1.391 (0.538) |
| Ridge | 10000 | **0.181 (0.046)** | **0.181 (0.051)** | **0.188 (0.063)** | **0.203 (0.080)** | **0.224 (0.101)** | **0.269 (0.149)** | **0.360 (0.225)** |

**eTable 4:** AUC values (empirical standard error) in the validation population. Maximum AUC values across each sample size are given in bold.

| **Model** | **Training set Sample Size** | **σ=0.00** | **σ=0.125** | **σ=0.250** | **σ=0.375** | **σ=0.500** | **σ=0.750** | **σ=1.00** |
| --- | --- | --- | --- | --- | --- | --- | --- | --- |
| PCA | 150 | 0.711 (0.009) | 0.707 (0.009) | 0.705 (0.009) | 0.704 (0.009) | 0.707 (0.009) | 0.723 (0.009) | 0.731 (0.008) |
| PLS | 150 | 0.716 (0.009) | 0.713 (0.009) | 0.710 (0.009) | 0.707 (0.009) | 0.708 (0.009) | 0.717 (0.009) | 0.723 (0.009) |
| SR | 150 | **0.721 (0.009)** | **0.717 (0.009)** | **0.714 (0.009)** | **0.710 (0.009)** | 0.711 (0.009) | 0.713 (0.009) | 0.711 (0.009) |
| AIC | 150 | 0.646 (0.010) | 0.646 (0.010) | 0.657 (0.010) | 0.669 (0.009) | 0.686 (0.009) | 0.719 (0.009) | 0.742 (0.008) |
| Ridge | 150 | 0.669 (0.009) | 0.671 (0.009) | 0.682 (0.009) | 0.696 (0.009) | **0.712 (0.009)** | **0.748 (0.008)** | **0.770 (0.008)** |
| PCA | 250 | 0.720 (0.009) | 0.714 (0.009) | 0.717 (0.009) | 0.713 (0.009) | 0.716 (0.009) | 0.732 (0.009) | 0.744 (0.008) |
| PLS | 250 | 0.721 (0.009) | 0.716 (0.009) | 0.718 (0.009) | 0.713 (0.009) | 0.713 (0.009) | 0.727 (0.009) | 0.738 (0.008) |
| SR | 250 | **0.726 (0.009)** | **0.720 (0.009)** | **0.721 (0.009)** | 0.715 (0.009) | 0.713 (0.009) | 0.716 (0.009) | 0.721 (0.009) |
| AIC | 250 | 0.680 (0.009) | 0.679 (0.009) | 0.695 (0.009) | 0.705 (0.009) | 0.722 (0.009) | 0.756 (0.008) | 0.784 (0.008) |
| Ridge | 250 | 0.692 (0.009) | 0.692 (0.009) | 0.708 (0.009) | **0.717 (0.009)** | **0.733 (0.009)** | **0.764 (0.008)** | **0.792 (0.008)** |
| PCA | 500 | 0.724 (0.009) | 0.723 (0.009) | 0.720 (0.009) | 0.720 (0.009) | 0.728 (0.009) | 0.740 (0.008) | 0.753 (0.008) |
| PLS | 500 | 0.724 (0.009) | 0.722 (0.009) | 0.720 (0.009) | 0.719 (0.009) | 0.725 (0.009) | 0.736 (0.009) | 0.749 (0.008) |
| SR | 500 | **0.727 (0.009)** | **0.725 (0.009)** | 0.721 (0.009) | 0.717 (0.009) | 0.720 (0.009) | 0.718 (0.009) | 0.722 (0.009) |
| AIC | 500 | 0.707 (0.009) | 0.712 (0.009) | 0.720 (0.009) | 0.734 (0.009) | 0.750 (0.008) | 0.786 (0.008) | **0.811 (0.007)** |
| Ridge | 500 | 0.710 (0.009) | 0.715 (0.009) | **0.724 (0.009)** | **0.737 (0.009)** | **0.753 (0.008)** | **0.787 (0.008)** | **0.811 (0.007)** |
| PCA | 1000 | 0.728 (0.009) | 0.726 (0.009) | 0.724 (0.009) | 0.728 (0.009) | 0.731 (0.009) | 0.741 (0.008) | 0.754 (0.008) |
| PLS | 1000 | 0.728 (0.009) | 0.725 (0.009) | 0.723 (0.009) | 0.727 (0.009) | 0.730 (0.009) | 0.739 (0.008) | 0.752 (0.008) |
| SR | 1000 | **0.729 (0.009)** | 0.726 (0.009) | 0.722 (0.009) | 0.722 (0.009) | 0.721 (0.009) | 0.718 (0.009) | 0.721 (0.009) |
| AIC | 1000 | 0.725 (0.009) | **0.727 (0.009)** | **0.736 (0.009)** | **0.752 (0.008)** | **0.767 (0.008)** | **0.796 (0.008)** | **0.820 (0.007)** |
| Ridge | 1000 | 0.724 (0.009) | **0.727 (0.009)** | **0.736 (0.009)** | **0.752 (0.008)** | 0.766 (0.008) | 0.795 (0.008) | 0.818 (0.007) |
| PCA | 5000 | 0.731 (0.009) | 0.730 (0.009) | 0.728 (0.009) | 0.731 (0.009) | 0.736 (0.009) | 0.747 (0.008) | 0.760 (0.008) |
| PLS | 5000 | 0.731 (0.009) | 0.730 (0.009) | 0.728 (0.009) | 0.730 (0.009) | 0.735 (0.009) | 0.747 (0.008) | 0.760 (0.008) |
| SR | 5000 | 0.730 (0.009) | 0.728 (0.009) | 0.725 (0.009) | 0.723 (0.009) | 0.723 (0.009) | 0.724 (0.009) | 0.723 (0.008) |
| AIC | 5000 | **0.736 (0.009)** | **0.741 (0.009)** | **0.751 (0.008)** | **0.763 (0.008)** | **0.778 (0.008)** | **0.807 (0.007)** | **0.832 (0.007)** |
| Ridge | 5000 | **0.736 (0.009)** | 0.740 (0.009) | 0.750 (0.008) | 0.762 (0.008) | 0.777 (0.008) | 0.806 (0.007) | 0.831 (0.007) |
| PCA | 10000 | 0.732 (0.009) | 0.729 (0.009) | 0.730 (0.009) | 0.732 (0.009) | 0.733 (0.009) | 0.751 (0.008) | 0.764 (0.008) |
| PLS | 10000 | 0.732 (0.009) | 0.729 (0.009) | 0.730 (0.009) | 0.732 (0.009) | 0.733 (0.009) | 0.751 (0.008) | 0.764 (0.008) |
| SR | 10000 | 0.731 (0.009) | 0.727 (0.009) | 0.726 (0.009) | 0.724 (0.009) | 0.720 (0.009) | 0.725 (0.009) | 0.726 (0.009) |
| AIC | 10000 | **0.739 (0.009)** | **0.742 (0.009)** | **0.753 (0.008)** | **0.766 (0.008)** | **0.778 (0.008)** | **0.811 (0.007)** | **0.834 (0.007)** |
| Ridge | 10000 | 0.738 (0.009) | **0.742 (0.009)** | **0.753 (0.008)** | **0.766 (0.008)** | 0.777 (0.008) | 0.810 (0.007) | 0.833 (0.007) |

# Supplementary References

1. Aguilera AM, Escabias M, Valderrama MJ: **Using principal components for estimating logistic regression with high-dimensional multicollinear data**. *Comput Stat Data Anal* 2006, **50**:1905–1924.

2. Hastie, Trevor, Tibshirani, Robert, Friedman J: *The Elements of Statistical Learning The Elements of Statistical LearningData Mining, Inference, and Prediction, Second Edition*. 2009.

# R Code

## Main Simulation Function

Simulation.fn <- function(iter=100, CandidateSize, DevSampleSize , ValSampleSize=5000, M, true.risks.lower, true.risks.upper, P, true.model.which, P.X, SigmaVals=0, sigma0=0, ProbY=0.5, CorrCluster, rho, ProbTrue, ProbMiss, filename, workingDirec, StartingSeed=4359){
 #Input: iter = the number of iterations to run the simulation over- defaults at 100
 # CandidateSize = number of observations in each candidate model population. Supply either as a scalar giving the same number of observations in each population, or a vector (same length as M) giving number of observations in each candidate model population.
 # ValSampleSize = a scalar giving the number of samples to validate new models on - defaults as 5000
 # DevSampleSize = a vector giving the development population sizes to run the simulation over.
 # M = number of candidate models to simulate.
 # true.risks.lower/ true.risks.upper = lower/ upper bounds on the "true" risk coefficients.
 # P = number of covariates to generate.
 # true.model.which = a vector with binary entries indicating which of the P covariates are included in the true model (exclude the intercept since this is always included in the models).
 # P.X = a vector (length same as number of parameters) with entries indicating if the jth parameter is continuous (NA) or binary (cut-off value). If cut-off values are given, give the event probability of that variable (i.e. P(X_j=1)).
 # SigmaVals = a vector giving the population level variation in 'true' risks to run the simulation over.
 # sigma0 = a scalar giving the varation in overall event rates over each population for all simulations- defaults at zero.
 # ProbY = a scalar giving the average probability of Y=1 before population level variation (sigma0)- defaults to 0.5.
 # CorrCluster = a scalar giving the number of parameters in each cluster of serially correlated variables (must be greater than 2).
 # rho = the degree of correlation between predictors in each cluster.
 # ProbMiss = a matrix (nrows = M ncol= num of clusters) giving the probability a candidate model misses all variables within each cluster.
 # ProbTrue = a matrix (nrows = M ncol= num of clusters) giving the probability a candidate model includes both a 'true' predictor in addition to a correlated predictor in each cluster.
 # filename = character variable giving the name of the text file to store the results.
 # StartingSeed = number giving the seed to set at the very start of the simulation. Defaults at 4359, which was the seed used for the result given in this paper.

 set.seed(StartingSeed)

 require(pROC) #discrimination package.
 require(glmnet) #used to fit the penalized logistic regression.
 require(plsRglm) #Partial Least Square for logistic regression.
 require(scoring) #used to calculate the Brier Score.
 require(dplyr) #data manipulation.


 #Create text file to store all the performance results.
 if(file.exists(paste(filename, ".txt", sep=""))==TRUE){
 #stops from accidentally overwriting a results file.
 stop("filename already exists - delete or move file to continue")#.
 }else{
 filename <- paste(filename, ".txt", sep="")
 OutputNames <- c("Iteration", "Model", "SampleSize", "Sigma",
 "CalInter", "SE.CalInter",
 "CalSlope", "SE.CalSlope",
 "AUC", "SE.AUC",
 "MSE_Predictions",
 "MSE_Parameters",
 "MeanGenCoefBinaryDiff",
 "MeanGenCoefContinuousDiff",
 "SDGenBinaryCoef",
 "SDGenContinuousCoef")
 #Create the output file with the above column headings.
 write(OutputNames, filename, append=FALSE, sep="|", ncolumns=length(OutputNames))
 }

 for(Devsample in DevSampleSize){ #across all sample sizes.
 for(sigma in SigmaVals){ #across all sigma values.
 pb <- winProgressBar(title="Simulation Progress bar", label=paste("Simulation 0% Completed"), min=0, max=100, initial=0)

 if(length(CandidateSize)==1){
 n.obs <- c(rep(CandidateSize, M), (Devsample+ValSampleSize))
 }else if(length(CandidateSize)==M){
 n.obs <- c(CandidateSize, (Devsample+ValSampleSize))
 }else{
 stop("CandidateSize must either be single value or vector of size M")
 }

 for(i in 1:iter){ #across all iterations.
 # candidate.model.predictors.fn returns a M by P matrix with binary entries indicating if the pth parameter is included in model m.
 include.para <- candidate.model.predictors.fn(M=M, P=P, CorrCluster=CorrCluster, ProbTrue=ProbTrue, ProbMiss=ProbMiss, true.model.which=true.model.which)

 #Generate datasets and response variables in each population.
 Combined.Data.call <- Combined.data.fn(n.obs=n.obs, M=M, true.risks.lower=true.risks.lower, true.risks.upper=true.risks.upper, P=P, true.model.which=true.model.which, P.X=P.X, sigma=sigma, sigma0=sigma0, ProbY=ProbY, CorrCluster=CorrCluster, rho=rho) #.

 Combined.Data <- Combined.Data.call$Combined.Data %>%
 select(Population, id, everything()) #Extract the data with covariates and binary response variables, then move population and ID to the left of the dataset (no effect on modelling).

 #Extract the generating coefficients for all parameters and place into data.frame.
 GeneratingCoefs <- Combined.Data.call$GenCoefs
 colnames(GeneratingCoefs) <- names(Combined.Data %>% select(Intercept, starts_with("V")))
 GeneratingCoefs <- data.frame(GeneratingCoefs)

 #Extract the development cohort randomly.
 train.test.pop <- Combined.Data %>% filter(Population==(M+1))
 test_ind <- sort(sample(train.test.pop$id, size = ValSampleSize))
 Combined.Data$Population[which(Combined.Data$id%in%test_ind)] <- (M+2)
 rm(train.test.pop, test_ind)#.

 #Run the modelling function with the combined data, which fits the M existing models (each with variables determined by the matrix “include.para”), fits the aggregate models and fits two new models.
 Models <- Modelling.fn(Combined.Data, M=M, Include.para= include.para)

 #Combined.Data now includes the predicted risks and linear predictors from each model.
 Combined.Data <- Models$Combined.Data

 Beta <- Models$Beta.Mat

 #Extract the samples serving as the validation population.
 TestData <- Combined.Data %>% filter(Population==(M+2))


 #Candidate model performance in population of interest.
 PerformanceCandidate <- t(sapply(TestData%>%select(starts_with("PredRisk")), mod.perform.fn, Outcomes=TestData$Y, TrueRisks=TestData$GeneratingRisk))

 #Performance of PCA model.
 PerformancePCA <- mod.perform.fn(TestData$PCA.PredRisk, Outcomes=TestData$Y, TrueRisks=TestData$GeneratingRisk)

 #Performance of PLS model.
 PerformancePLS <- mod.perform.fn(TestData$PLS.PredRisk, Outcomes=TestData$Y, TrueRisks=TestData$GeneratingRisk)

 #Performance of Stacked regression model.
 PerformanceSR <- mod.perform.fn(TestData$SR.Original.PredRisk, Outcomes=TestData$Y, TrueRisks=TestData$GeneratingRisk)

 #AIC Re-developed model performance.
 PerformanceNew <- mod.perform.fn(TestData$NewPredRisks, Outcomes=TestData$Y, TrueRisks=TestData$GeneratingRisk)

 #Ridge Re-developed model performance.
 PerformanceRidge <- mod.perform.fn(TestData$Ridge.pred, Outcomes=TestData$Y, TrueRisks=TestData$GeneratingRisk)

 #Performance of the Generating Model.
 PerformanceGenerating <- mod.perform.fn(TestData$GeneratingRisk, Outcomes=TestData$Y, TrueRisks=TestData$GeneratingRisk)

 #Store the performance of all models.
 Performance <- data.frame(rbind(PerformanceCandidate,
 PerformancePCA,
 PerformancePLS,
 PerformanceSR,
 PerformanceNew,
 PerformanceRidge,
 PerformanceGenerating)) %>%
 mutate(Model=c(paste("Original", 1:M, sep=""),
 "PCA",
 "PLS",
 "SR",
 "AIC",
 "Ridge",
 "Generating"),
 Iteration = rep(i, nrow(.)),
 SampleSize = Devsample,
 Sigma = sigma) %>%
 select(Iteration, Model, SampleSize, Sigma, everything())

 #Calculate the mean squared differences between each model coefficient and the generating coefficient in the dev. population.
 MSE.Parameters <- rep(NA, (M+5))
 for(m in 1:(M+5)){
 MSE.Parameters[m] <- ((1/(P+1))*(sum((Beta[m,]-GeneratingCoefs[(M+1),])^2)))
 }
 MSE.Parameters <- c(MSE.Parameters, 0) #clearly the mean squared error of the generating model is zero.


 NonZeroGenCoef <- GeneratingCoefs %>% select(-Intercept) %>%
 select(which(colSums(.)!=0)) #Extract all non-zero coefficients across all populations.

 NumUnique <- apply(select(Combined.Data, which(names(Combined.Data)%in%names(NonZeroGenCoef))),
 2, function(x)length(unique(x)))
 CoefType <- ifelse(NumUnique==2, "Binary", "Continuous") #Indicate which coefficient is continuous and which binary.

 #Add the sum square errors (MSD), the mean difference in generating coefficients and the standard deviation of the model parameters to the performance matrix. This was done for continuous and binary variable separately.
 Performance <- Performance %>%
 mutate(MSE_Parameters = MSE.Parameters,
 MeanGenCoefBinaryDiff = mean(apply(NonZeroGenCoef[,which(CoefType=="Binary")],2,max)-apply(NonZeroGenCoef[,which(CoefType=="Binary")],2,min)),
 MeanGenCoefContinuousDiff = mean(apply(NonZeroGenCoef[,which(CoefType=="Continuous")],2,max)-apply(NonZeroGenCoef[,which(CoefType=="Continuous")],2,min)),
 SDGenBinaryCoef = mean(apply(NonZeroGenCoef[,which(CoefType=="Binary")], 2, sd)),
 SDGenContinuousCoef = mean(apply(NonZeroGenCoef[,which(CoefType=="Continuous")], 2, sd)))

 rm(NumUnique, CoefType)#.

 #Write the results of this iteration to the results text file.
 write.table(Performance, filename, append=TRUE, sep="|", row.names=FALSE, col.names=FALSE)

 #Alter the progress bar.
 info <- sprintf(paste("Simulation %d%% Completed"), round((i/(iter)*100)))
 setWinProgressBar(pb, i/((iter))*100, label=info)
 }
 close(pb)
 }
 print(paste("Simulation of sample size", Devsample, "completed across all sigma", sep=" "))
 }
}

## Data Generation Functions

The following functions are called from the main function as part of data-generation procedure.

#The below function accepts probabilities of missing variables or including generating predictors and returns a matrix (of dim M by P) giving binary indicators if the pth paramter is included in model m..
candidate.model.predictors.fn <- function(M, P, CorrCluster, ProbTrue, ProbMiss, true.model.which){
 models.which <- matrix(NA, nrow=M, ncol=P, byrow=TRUE)#.
 for(m in 1:M){
 for(k in 1:ceiling(P/CorrCluster)){ #For current m, determine which predictors are included in each cluster.
 ClusterColumnNumbers <- (((k-1)*(CorrCluster)+1):(k*CorrCluster)) #a sequence of columns numbers in cluster k.

 P_Miss <- rbinom(1, 1, ProbMiss[m,k])
 if(P_Miss==1){ #Model m misses all predictors in this cluster.
 models.which[m, ClusterColumnNumbers] <- 0
 }else{
 P_True <- rbinom(1, 1, (ProbTrue[m,k]/(1-ProbMiss[m,k]))) #adjust the probability to account for P(Miss).

 models.which[m, ClusterColumnNumbers] <- 0
 if(P_True==1){ #then model m includes a true predictor AND a correlated predictor in this cluster.
 models.which[m, ((ClusterColumnNumbers)[which(true.model.which[ClusterColumnNumbers]!=0)])] <- 1
 models.which[m, ClusterColumnNumbers][sample(2:CorrCluster,1, replace=FALSE)] <- 1 #randomly include a correlated predictors in cluster.
 }else{ #Else model M only includes a correlated predictor.
 models.which[m, ClusterColumnNumbers][sample(2:CorrCluster,1, replace=FALSE)] <- 1 #randomly include a correlated predictors in cluster.
 }
 }
 rm(ClusterColumnNumbers)
 }
 }
 return(models.which)
}


#The below function returns a generated dataset with the design matrix X, outcomes, Y and generating predicted probabilities.
Combined.data.fn <- function(n.obs, M, true.risks.lower, true.risks.upper, P, true.model.which, P.X, sigma, sigma0, ProbY, CorrCluster, rho){

 N <- sum(n.obs) #Total number of observations to sample.

 Cutoffs <- qnorm(P.X)

 X <- matrix(NA, nrow=N, ncol=P) #a matrix to store the predictors.
 Temp.X <- matrix(NA, nrow=N, ncol=P) #matrix to store latent normally distributed variables before being dichotomized.

 for(p in 1:P){
 if(is.na(Cutoffs[p])){ #if no cut-off value is given for parameter p, keep as a continuous variable .
 if(((p-1)%%(CorrCluster))==0){ #if p is the 'start' of a new cluster, then simulate the normal independently.
 X[,p] <- rnorm(n=N, mean=0, sd=1) #simulate (without loss of generality) from the standard normal.

 }else{ #else simulate based on the correlation between terms within the clusters.
 Z <- rnorm(n=N, mean=0, sd=1)
 X[,p] <- (rho*X[,p-1]) + ((sqrt(1-(rho^2)))*Z)
 }
 }else{ #if cut-off value is given for parameter p, we simulate a binary variable as a latent normal.
 if(((p-1)%%(CorrCluster))==0){ #if p is the 'start' of a new cluster, then simulate the normal independently.
 Temp.X[,p] <- rnorm(n=N, mean=0, sd=1) #simulate (without loss of generality) from the standard normal.

 }else{ #else simulate based on the correlation between terms within the clusters.
 u <- runif(N, 0,1) #generate a probability uniformly.
 Z <- rnorm(n=N, mean=0, sd=1)
 Temp.X[,p] <- ifelse(u<=rho, Temp.X[,p-1], Z) #if the probability is < rho then same as previous parameter.
 }
 X[,p] <- ifelse(test=Temp.X[,p]<Cutoffs[p], yes=1, no=0) #turn to binary variable based on pre-defined cut-off values.
 }
 }

 X <- cbind(rep(1, nrow(X)), X) #Add intercept to design matrix, X
 rm(Temp.X) #remove the temporary X matrix
 colnames(X) <- c("Intercept", paste("V", 1:P, sep=""))

 X <- data.frame(X) %>%
 mutate(Population = rep(1:(M+1), times=n.obs),
 id = seq(from=1, to=N, by=1))

 n.variables <- ncol(X %>% select(starts_with("V")))

 alpha <- rep(0, n.variables)#.
 alpha[which(true.model.which!=0)] <- runif(length(which(true.model.which!=0)), true.risks.lower, true.risks.upper) #sample the over-arching mean generating coefficients.

 #Apply random variation to the mean generating coefficients in each population.
 alpha.pop <- t(replicate((M+1), alpha))
 alpha.pop[, colSums(alpha.pop != 0) > 0] <- apply(alpha.pop[, colSums(alpha.pop != 0) > 0], 2, function(x) x+rnorm(length(x), 0, sigma))
 alpha.pop <- cbind(rep(NA, nrow(alpha.pop)), alpha.pop)

 Q.x <- NULL
 for(m in 1:(M+1)){
 XmatpopNOinter <- X %>%
 filter(Population==m) %>%
 select(starts_with("V")) %>%
 data.matrix()#.

 #set the true model intercept so the event rate of Y matches that desired by the user.
 alpha0 <- as.numeric(coef(glm(rbinom(dim(XmatpopNOinter)[1], 1, ProbY)~offset(XmatpopNOinter%*%alpha.pop[m,-1]), family=binomial(link="logit"))))
 alpha0 <- alpha0+rnorm(1, 0, sigma0)#.

 alpha.pop[m,1] <- alpha0
 rm(XmatpopNOinter)

 Xmatpop <- X %>%
 filter(Population==m) %>%
 select(Intercept, num_range("V", 1:(n.variables))) %>%
 data.matrix()

 Q.x.temp <- ( (exp(Xmatpop%*%alpha.pop[m,]))/(1+(exp(Xmatpop%*%alpha.pop[m,]))) )

 Q.x <- c(Q.x, Q.x.temp)
 rm(Q.x.temp); rm(Xmatpop)
 }
 #Generate binary responses based on the probability obtained from the generating model in each population.
 y <- rbinom(n=nrow(X), size=1, prob=Q.x)
 Y <- data.frame("Population" = X$Population,
 "id" = X$id,
 "GeneratingRisk" = Q.x,
 "Y"=y)
 #Merge the generated binary outcomes and the generating risks with the parameters by id and population.
 Combined.Data <- X %>%
 left_join(Y, by=c("id", "Population"))

 return(list("Combined.Data"=Combined.Data,
 "GenCoefs"=alpha.pop))

}

#

## Modelling Functions

#The below function takes the generated dataset and models each candidate model, the aggregate models and the newly derived models.
Modelling.fn <- function(Combined.Data, M, Include.para){

 P <- ncol(Include.para) #The total number of parameters.

 Beta.Mat <- matrix(NA, nrow=(M+5), ncol=(P+1)) #will store estimated coefficients in each model.

 #Fit the existing models to each existing population (calls the function “Model.fn” which is given below).
 CandidateModels <- lapply(1:M, function(x)Model.fn(Data=Combined.Data[which(Combined.Data$Population==x),], Included.Parameters=which(Include.para[x,]!=0)))

 PredRisks <- sapply(CandidateModels, function(x)predict(x, newdata=Combined.Data, type="response")) #obtained predicted risks from each candidate model.
 PredRisks <- data.frame(PredRisks, "id"=as.numeric(row.names(PredRisks))) #store predicted risks in a data.frame.
 names(PredRisks) <- c(paste("PredRisk", 1:M, sep=""), "id")

 LinearPredictors <- sapply(CandidateModels, function(x)predict(x, newdata=Combined.Data, type="link")) #obtained linear predictors from each candidate model
 LinearPredictors <- data.frame(LinearPredictors, "id"=as.numeric(row.names(LinearPredictors))) # store linear predictors in a data.frame.
 names(LinearPredictors) <- c(paste("LP", 1:M, sep=""), "id")

 # merge the predicted risks and linear predictors obtained from each candidate model with the combined data (i.e. X and Y).
 Combined.Data <- Combined.Data %>%
 left_join(PredRisks, by="id") %>%
 left_join(LinearPredictors, by="id")

 for(m in 1:M){
 Beta.Mat[m, c(1, (which(Include.para[m, ] == 1)+1))] <- as.numeric(coef(CandidateModels[[m]]))
 Beta.Mat[m, which(is.na(Beta.Mat[m,]))] <- 0 #record and store the coefficient values for each candidate CPM.
 }

 Design.Mat.Dev <- Combined.Data %>%
 filter(Population==(M+1))

 #### Now aggregate or re-develop a model in the (local) population.

 #Principal Component Analysis Model.
 LP <- Combined.Data %>%
 filter(Population==(M+1)) %>%
 select(num_range("LP", 1:M)) #Linear predictors from each candidate model in the training sample.

 pca <- prcomp(LP, scale = FALSE, center = FALSE, retx=TRUE)
 PC <- data.frame(pca$x) #the matrix of principal components (LP matrix multiplied by the pca loading matrix).

 mod <- glm(Design.Mat.Dev$Y ~., data=PC, family=binomial(link="logit")) #Full Model.
 null.mod <- glm(Design.Mat.Dev$Y ~PC1, data=PC, family=binomial(link="logit")) #Null model - keep at least the 1st PC.
 PCA.mod <- step(mod, scope=list(lower=null.mod, upper=mod), direction="backward", trace=FALSE) #Backwards selection using AIC.

 PCA.Coefs <- matrix(0, nrow=1, ncol=(M+1)) #Extract the PCA model coefficients.
 colnames(PCA.Coefs) <- c("(Intercept)", paste("PC", 1:M, sep="")) #Extract the PCA model coefficients.
 PCA.Coefs[,names(coef(PCA.mod))] <- coef(PCA.mod) #Extract the PCA model coefficients.

 Loading.mat <- pca$rotation #Rotation/loading matrix from PCA.

 #Fit the PCA model to the whole dataset.
 LP.all <- Combined.Data %>%
 select(num_range("LP", 1:M))
 new.dat <- data.frame(predict(pca, newdata=LP.all)) #Principal components for all samples.
 names(new.dat) <- names(PC)

 PCA.PredRisk <- predict(PCA.mod, newdata=new.dat, type="response") #the predicted risks from the PCA model.
 PCA.PredRisk <- data.frame("PCA.PredRisk"=PCA.PredRisk, "id"=1:length(PCA.PredRisk))
 names(PCA.PredRisk) <- c("PCA.PredRisk", "id")

 # merge the PCA predicted risks with the combined data (i.e. X and Y).
 Combined.Data <- Combined.Data %>%
 left_join(PCA.PredRisk, by="id")

 #Transform the PCA component coefficients onto the scale of the original risk factors in each model.
 zeta <- c(as.numeric(PCA.Coefs[1,1]),c(Loading.mat%*%PCA.Coefs[,-1]))
 for(p in 1:(P+1)){
 if(p==1){
 Beta.Mat[M+1, p] <- (zeta[1]+ (zeta[-1]%*%Beta.Mat[1:M, p]))
 }else{
 Beta.Mat[M+1, p] <- (zeta[-1]%*%Beta.Mat[1:M, p])
 }
 }


 #Partial Least Squares Model.
 PLS.mod <- plsRglm(dataY=Design.Mat.Dev$Y, dataX=LP, nt=M, modele="pls-glm-logistic", scaleX = FALSE, scaleY = FALSE, verbose=FALSE, sparse=TRUE, sparseStop=TRUE) #.

 # Store the coefficients on the original scale from PLS.
 PLS.coefficients <- as.numeric(coef(PLS.mod, type="original")$Coeffs)
 LP.all <- data.matrix(LP.all)
 PLS.design.mat <- cbind(rep(1,nrow(LP.all)), LP.all)

 # calculate the PLS predicted risks for all observations.
 PLS.PredRisk <- ( (exp(PLS.design.mat%*%PLS.coefficients)) / (1+(exp(PLS.design.mat%*%PLS.coefficients))) )
 PLS.PredRisk <- data.frame("PLS.PredRisk"=PLS.PredRisk, "id"=1:length(PLS.PredRisk))
 names(PLS.PredRisk) <- c("PLS.PredRisk", "id")

 # merge the PLS predicted risks with the combined data (i.e. X and Y).
 Combined.Data <- Combined.Data %>%
 left_join(PLS.PredRisk, by="id")

 PLS.coefficients <- as.numeric(coef(PLS.mod, type="original")$Coeffs)
 #Transform the PLS coefficients onto the scale of the original risk factors in each model.
 for(p in 1:(P+1)){
 if(p==1){
 Beta.Mat[M+2, p] <- (PLS.coefficients[1]+
 (PLS.coefficients[-1]%*%Beta.Mat[1:M, p]))
 }else{
 Beta.Mat[M+2, p] <- (PLS.coefficients[-1]%*%Beta.Mat[1:M, p])
 }
 }


 #Stacked Regression.
 SR.Original <- Stacked.Regression(LinPreds=Combined.Data[which(Combined.Data$Population==(M+1)), paste("LP", 1:M, sep="")], M=M, Outcome=Combined.Data[which(Combined.Data$Population==(M+1)),"Y"])

 SR.Mat <- Combined.Data %>%
 select(Intercept, num_range("LP", 1:M)) %>% data.matrix()

 # obtain predicted risks from the SR model.
 SR.Original.PredRisk.Val <- SR.Mat%*%SR.Original$Weights
 SR.Original.PredRisk.Val <- exp(SR.Original.PredRisk.Val)/(1+exp(SR.Original.PredRisk.Val))
 SR.Original.PredRisk.Val <- data.frame("SR.Original.PredRisk"=SR.Original.PredRisk.Val, "id"=1:nrow(SR.Original.PredRisk.Val))

 # merge the SR predicted risks with the combined data (i.e. X and Y).
 Combined.Data <- Combined.Data %>%
 left_join(SR.Original.PredRisk.Val, by="id")

 StackedRegCoefs <- SR.Original$Weights #coefficients from the stacked regression model.
 #Transform the SR weights onto the scale of the original risk factors in each model.
 for(p in 1:(P+1)){
 if(p==1){
 Beta.Mat[M+3, p] <- (StackedRegCoefs[1]+
 (StackedRegCoefs[-1]%*%Beta.Mat[1:M, p]))
 }else{
 Beta.Mat[M+3, p] <- (StackedRegCoefs[-1]%*%Beta.Mat[1:M, p])
 }
 }


 #Model re-development.
 Design.Mat.Dev <- Combined.Data %>%
 filter(Population==(M+1)) %>%
 select(Y, Intercept, num_range("V", unique(col(Include.para)[which(Include.para!=0)])))

 full.model <- glm(Y ~-1+., data=Design.Mat.Dev, family=binomial(link="logit"))
 Derived.Model <- step(full.model, direction = "backward", trace=FALSE) #Backwards selection using AIC.

 Design.Mat.All <- Combined.Data %>%
 select(Intercept, num_range("V", unique(col(Include.para)[which(Include.para!=0)])))

 New.pred <- predict(Derived.Model, newdata=Design.Mat.All, type="response")
 New.pred <- data.frame("NewPredRisks"=New.pred, "id"=1:length(New.pred))
 names(New.pred) <- c("NewPredRisks", "id")

 # merge the predicted risks from AIC model with the combined data (i.e. X and Y).
 Combined.Data <- Combined.Data %>%
 left_join(New.pred, by="id")

 #Store coefficients from the new derived model.
 Beta.Mat[M+4, ] <- 0
 Beta.Mat[M+4, match(names(coef(Derived.Model)),names(Combined.Data))] <- coef(Derived.Model)


 #Model re-development by Ridge regression.
 Design.Mat.Dev <- Combined.Data %>%
 filter(Population==(M+1)) %>%
 select(Y, num_range("V", unique(col(Include.para)[which(Include.para!=0)])))

 Variables <- Design.Mat.Dev %>%
 select(num_range("V", unique(col(Include.para)[which(Include.para!=0)]))) %>%
 data.matrix()

 Ridge.Model <- cv.glmnet(x=Variables, y=Design.Mat.Dev$Y, family="binomial", alpha=0)

 # obtained the predicted risks from the ridge regression model.
 Design.Mat.All <- Combined.Data %>%
 select(num_range("V", unique(col(Include.para)[which(Include.para!=0)]))) %>%
 data.matrix()

 Ridge.pred <- predict(Ridge.Model, newx=Design.Mat.All, type="response", s="lambda.min")
 Ridge.pred <- data.frame("Ridge.pred"=Ridge.pred, "id"=1:length(Ridge.pred))
 names(Ridge.pred) <- c("Ridge.pred", "id")

 # merge the ridge regression predicted risks with the combined data (i.e. X and Y).
 Combined.Data <- Combined.Data %>%
 left_join(Ridge.pred, by="id")

 #Store coefficients from the new derived model.
 Beta.Mat[M+5, ] <- 0
 Beta.Mat[M+5, c(1, (unique(col(Include.para)[which(Include.para!=0)])+1))] <- as.numeric(coef(Ridge.Model, s="lambda.min"))


 return(list("Combined.Data"=Combined.Data,
 "Beta.Mat"=Beta.Mat))
}


#The below function is called by "Modelling.fn" and models each candidate model based on the parameters indicated by “Included.Parameters” which is generated from the function "candidate.model.predictors.fn".
Model.fn <- function(Data, Included.Parameters){

 Model.dgn.mat <- Data %>%
 select(Y, num_range("V", Included.Parameters))

 mdls <- glm(Y ~., data=Model.dgn.mat, family=binomial(link="logit"))

 return(mdls)

}

## Stacked Regression Functions

The below functions are used to maximise the logistic regression likelihood for stacked regression

#Logistic likelihood function for stacked regression.
likelihood.fn <- function(W, y, LP){
 #input: W = the vector of weights for the stacked regression
 # LP = the matrix of linear predictors from each simulated model
 # y = the vector of outcomes

 SR <- LP%*%W

 joint <- sum(-(y%*%log(1+exp(-SR)))-((1-y)%*%log(1+exp(SR)))) #log-Likelihood of logistic regression.

 return(-(joint)) #optim minimises and so return minus log-likelihood in order to maximise.
}


#Function to fit the stacked regression model.
Stacked.Regression <- function(LinPreds, M, Outcome){
 #input: LinPreds = the matrix of linear predictors from each simulated model.
 # M = the number of models which have been simulated.
 # Outcome = the vector of binary outcomes.

 bl <- c(-Inf, rep(0.00000001,M)) #lower bound on parameters - intercept is un-restricted, other terms are non-negative.
 bu <- c(rep(Inf,M+1)) #upper bound on parameters
 start <- c(0, rep(1/M, M)) #initial guess for weights- each model assigned equal weighting.

 if(!is.matrix(LinPreds)){
 LinPreds <- data.matrix(LinPreds)
 }

 LinPreds <- cbind(rep(1, dim(LinPreds)[1]), LinPreds) #Add intercept into the design matrix.
 MLE <- optim(start, likelihood.fn, y=Outcome, LP=LinPreds, method="L-BFGS-B", lower=bl, upper=bu, hessian=FALSE) #Optimise the likelihood function under parameter restrictions.

 W <- MLE$par #parameter estimates.
 W <- ifelse(abs(W)<0.000001, 0, W) # if returned weights are small, set them to zero.

 StackedRegression.Model <- glm(Outcome~ -1 + offset(I(LinPreds%*%W)), family=binomial(link="logit"))

 FittedRisks <- fitted(StackedRegression.Model)
 return(list("Weights"=W, "SR.PredictedRisks"=FittedRisks))
}

## Model Performance Function

The function “mod.perform.fn” assess the performance of all models in the validation sample

mod.perform.fn <- function(PredRisks, Outcomes, TrueRisks){

 Perform <- rep(NA, 7)
 # calibration intercept.
 calinter <- glm(Outcomes~offset(log(PredRisks/(1-PredRisks))), family=binomial(link="logit"))
 # calibration slope.
 calslope <- glm(Outcomes~(log(PredRisks/(1-PredRisks))), family=binomial(link="logit"))
 # AUC.
 discrim <- roc(response=Outcomes, predictor=PredRisks)
 # mean squared difference in the predicted risks against the generating risks
 MSE_Predictions <- mean((PredRisks-TrueRisks)^2)

 # store performance metrics and standard errors and return results.
 Perform[1] <- coef(calinter)[1]
 Perform[2] <- as.numeric(sqrt(diag(vcov(calinter))))
 Perform[3] <- coef(calslope)[2]
 Perform[4] <- as.numeric(sqrt(diag(vcov(calslope))))[2]
 Perform[5] <- as.numeric(discrim$auc)
 Perform[6] <- sqrt(var(discrim))
 Perform[7] <- MSE_Predictions

 return("Perform"=Perform)
}
